# Plume–MOR decoupling and the timing of India–Eurasia collision

**DOI:** 10.1038/s41598-022-16981-y

**Published:** 2022-08-03

**Authors:** Haytham Sehsah, Harald Furnes, Luan Thanh Pham, Ahmed M. Eldosouky

**Affiliations:** 1 Geology Department, Faculty of Science, Damietta University, New Damietta, Damietta, 34517 Egypt; 2 Department of Earth Science, University of Bergen, Allegt. 41, 5007 Bergen, Norway; 3 Department of Geophysics, Faculty of Physics, University of Science, Vietnam National University, Hanoi, Vietnam; 4 Geology Department, Faculty of Science, Suez University, Suez, 43518 Egypt

**Keywords:** Geochemistry, Tectonics

## Abstract

The debatable timing of India–Eurasia collision is based on geologic, stratigraphic, kinematic, and tectonic evidence. However, the collision event disturbed persistent processes, and the timing of disturbance in such processes could determine the onset of India–Eurasia collision precisely. We use the longevity of Southeast Indian Ridge (SEIR)—Kerguelen mantle plume (KMP) interaction cycles along the Ninetyeast ridge (NER) as a proxy to determine the commencement of India–Eurasia collision. The geochemical signature of the KMP tail along the NER is predominantly that of long-term coupling cycles, that was perturbed once by a short-term decoupling cycle. The long-term coupling cycles are mainly of enriched mid-ocean ridge basalts (E-MORBs). The short-term decoupling cycle is mostly derived from two distinct sources, MOR and plume separately, whereas the KMP is still being on-axis. The onset of India–Eurasia collision led to continental materials recycling into the mantle; hence the abrupt enrichment in incompatible elements at ca. 55 Ma, the MOR–plume on-axis decoupling, and the abrupt slowdown in the northward drift of the Indian plate was induced by the onset of India–Eurasia collision, thereafter MOR–plume recoupled.

## Introduction

**Figure 1:**
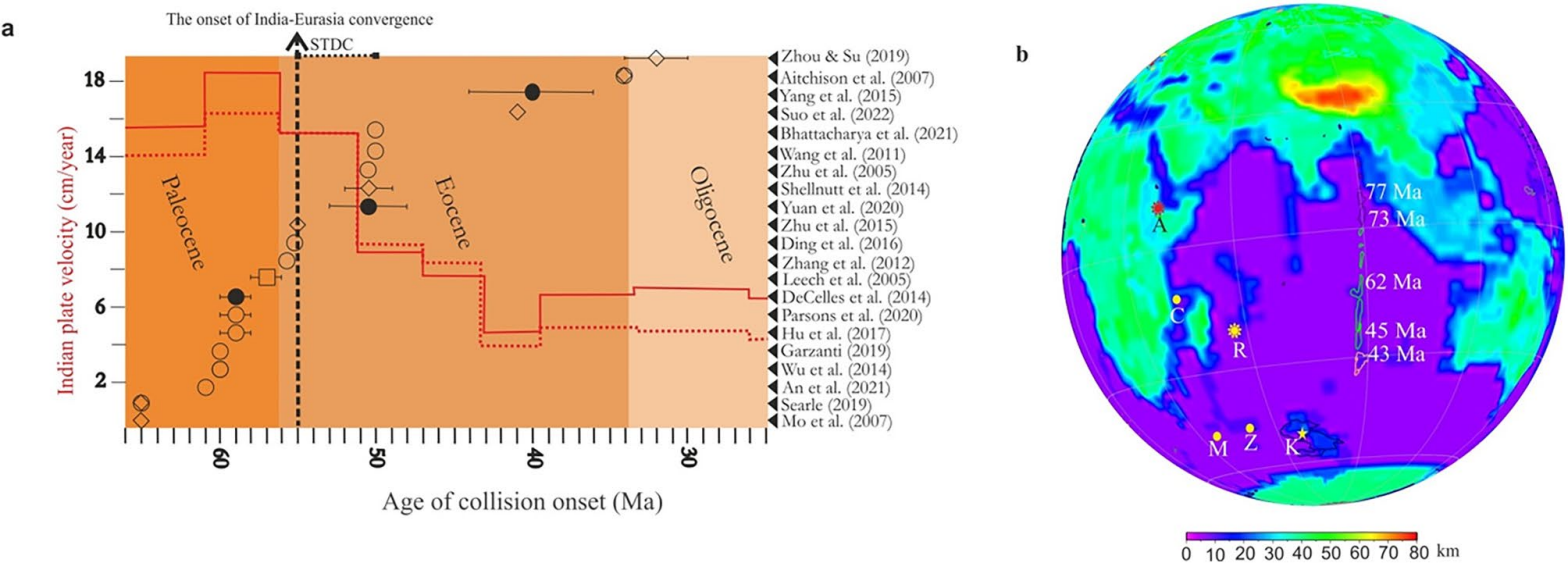
When did India–Eurasia collision start? (**a**) A broad spectrum of age possibilities^2–11,13,18,39–47^ based on different geologic, kinematic, and stratigraphic evidence; the velocity curve of the Indian plate in red after van Hinsbergen et al.^48^. Open circles, stratigraphy/sedimentary/provenance estimation; open diamonds, tecto-magmatic ages; squares, metamorphic clue; solid circles, paleomagnetic/kinematic evidence. (**b**) Large igneous provinces of the Ninetyeast ridge related to the KMP assigned to their ages in white following the linear age propagation equation of Pringle et al.^49^, with thick crust in the Indian Ocean^50^. *A* Afar, *C* Comoros, *K* Kerguelen, *M* Marion, *R* Reunion, *Z* Crozet.

The timing of India–Eurasia collision is debatable, and this is based on tecto-magmatic evidence, namely the cessation of Tethys crust subduction^1^, arc-magmatism episodes^2^, the rapid decrease in the northward drift of India^3^, age of ultrahigh-pressure metamorphism^4^, as well as stratigraphic^5–7^, paleomagnetic and geodynamic evidence^3,8,9^. Furthermore, the stratigraphic record is controversial, including the termination of the ocean pelagic sedimentation^10^, and bimodal sediments provenance reversal^5–7,9,11^. Thus, in-situ evidence for the timing of India–Eurasia collision have led to contrasting ages, ranging from 65 to 32 Ma (Fig. 1a). Subduction-related magmatism overlapped with the collision-related magmatism^12^, so the cessation of subduction magmatism cannot reconcile with the onset of the collision. Furthermore, a two-stage event of soft collision between India and the Tethyan microcontinents were followed by a two-stage event of hard collision between India and Eurasia; the quadruple stage of eastward collision happened diachronous from 52 to 38 Ma^13^. Meanwhile, the two-stage event of hard collision means that the onset of India–Eurasia collision happened twice, but there is no evidence for the recovery of the India plate velocity to the earlier rates to enable a second stage of hard collision (Fig. 1a). The rapid drift of the Indian plate was ascribed to plume head pushing and double-subduction pulling forces^14–16^, while the slowdown in the Indian plate drift was used as a clue for the initiation of collision^7,17^ . Meanwhile, the slowdown in the Indian plate drift has been challenged and explained as a result of the subducting Tethys lithosphere break-off rather than the initiation of India–Eurasia collision^18^. The discrepancy between geologic evidence leads to debatable timeframes for the emergence of India–Eurasia collision. Meanwhile, the spatial–temporal overlapping between such evidence gave a wide estimation error for the timing of that event. The stratigraphic age-constraints are mostly older, while tecto-magmatic ages are younger (Fig. 1a). Hence, we use the interaction between the Southeast Indian Ocean ridge (SEIR) and the Kerguelen mantle plume (KMP) along the Ninetyeast ridge (NER) as an alternative method for determining the onset of India–Eurasia collision.

Mantle plumes tend to elevate the upper mantle temperature underneath the slowly migrating mid-ocean ridge system (MORs), and this interaction stabilizes the MORs by extracting voluminous melts from the mantle^19^. Meanwhile, MOR–plume interaction persists for long periods by ridge jumps towards the mantle plume^20,21^. Furthermore, plumes flow towards MORs, boost the interaction between them by the plume capture mechanism, and this anchors the interaction due to ridge suction^19,22,23^. Nevertheless, when the plume is being off-axis, the distal interaction field between MORs and mantle plumes extends for distances greater than 1000 km^19,22^. The interaction between mantle plumes and the lithosphere is manifested in a dynamic topography^24–26^, large igneous provinces related to lithosphere breakup^19,27^, and the inherited older crustal materials in younger ridges^28^. The mantle plumes of the Comoros, Marion, Crozet, Kerguelen and Reunion plumes existed in the area once occupied by the amalgamated East Gondwana fragments^29,30^. Accordingly, large igneous provinces, and mantle plume tails related to mantle-plume activity are dominant in the Indian Ocean and within continents surrounding it^31^ (Fig. 1b). Furthermore, The KMP break-up East Antarctica and Australia obliquely to all orogenic structures^32,33^, one of the most puzzling elements of Pangaea supercontinent fragmentation. This process of semi-active rifting^34^ occurred in the presence of a small amount of syn-rift magma intrusion^35^. Regions of Precambrian continental crust recorded underneath the Indian Ocean are interpreted to be related to interactions with mantle plumes. Proterozoic garnet granulite xenoliths exists in the basaltic basement of Elan Bank related to the interaction with the KMP^36,37^, and inherited Archaean zircon found in Mauritius Island Miocene lava related to the Reunion plume^38^. Therefore, the kinematic of plates and the drift of the Indian plate is dependent on MOR–plume interaction, and in return the MOR–plume interaction is very sensitive to major tectonic events. Meanwhile, the diversity in melts extruded as a result of the interaction between the MORs and mantle plume records variations in mantle sources for the melts and give age-constraints for the tectonic processes.

We track the longevity of the plume–MOR interaction using the geochemical signature of the basaltic rocks along the NER to determine the sensitivity of plume–MOR coupling/decoupling cycles to the collision of India and Eurasia.

## ~ 55 Ma geochemical anomaly

Ocean island basalts (OIBs) are mainly derived from hot spots connected to a mantle plume^51^, while normal mid-ocean ridge basalts (N-MORBs) are extracted from depleted mantle and emplaced at MORs^52^. The interaction between MOR–plume rarely produces enriched mid-ocean ridge basalts (E-MORBs)^53^. However, E-MORBs emplaced along the NER were extruded as a result of interaction between the SEIR and KMP^54–56^, thus providing the geochemical signature that serves as a significant proxy in determining the longevity of the interaction between a MOR and mantle plume. Combinations of the little-mobile elements Th–Nb–Zr–Y–Yb define proxies that demonstrate the existence of different types of oceanic basalts, including N-MORB, E-MORB, and OIB, where the nature of these basalts are unrelated to subduction-related processes^57,58^. Thus, Th/Yb–Nb/Yb^57^, Nb/Y–Zr/Y^58^, and Th/Yb vs. Zr/Y^59^ diagrams are used to discriminate between 1164 (out of 6550) geochemical analyses for basalt volcanisms extruded along the NER in the Indian Ocean. Furthermore, normalized (La/Sm)_PM_ ratios to primitive mantle are used to remove the effect of magma differentiation processes. Sr–Nd–Pb–Hf isotopes are accompanied with trace element proxies to estimate the source of melts, the extent of MOR–plume interaction, and the involvement of crustal materials. The analyses along the NER are well-represented, and all the analyses are assigned to their ages (Supplementary Table 1).

### Samples and their age

**Figure 2:**
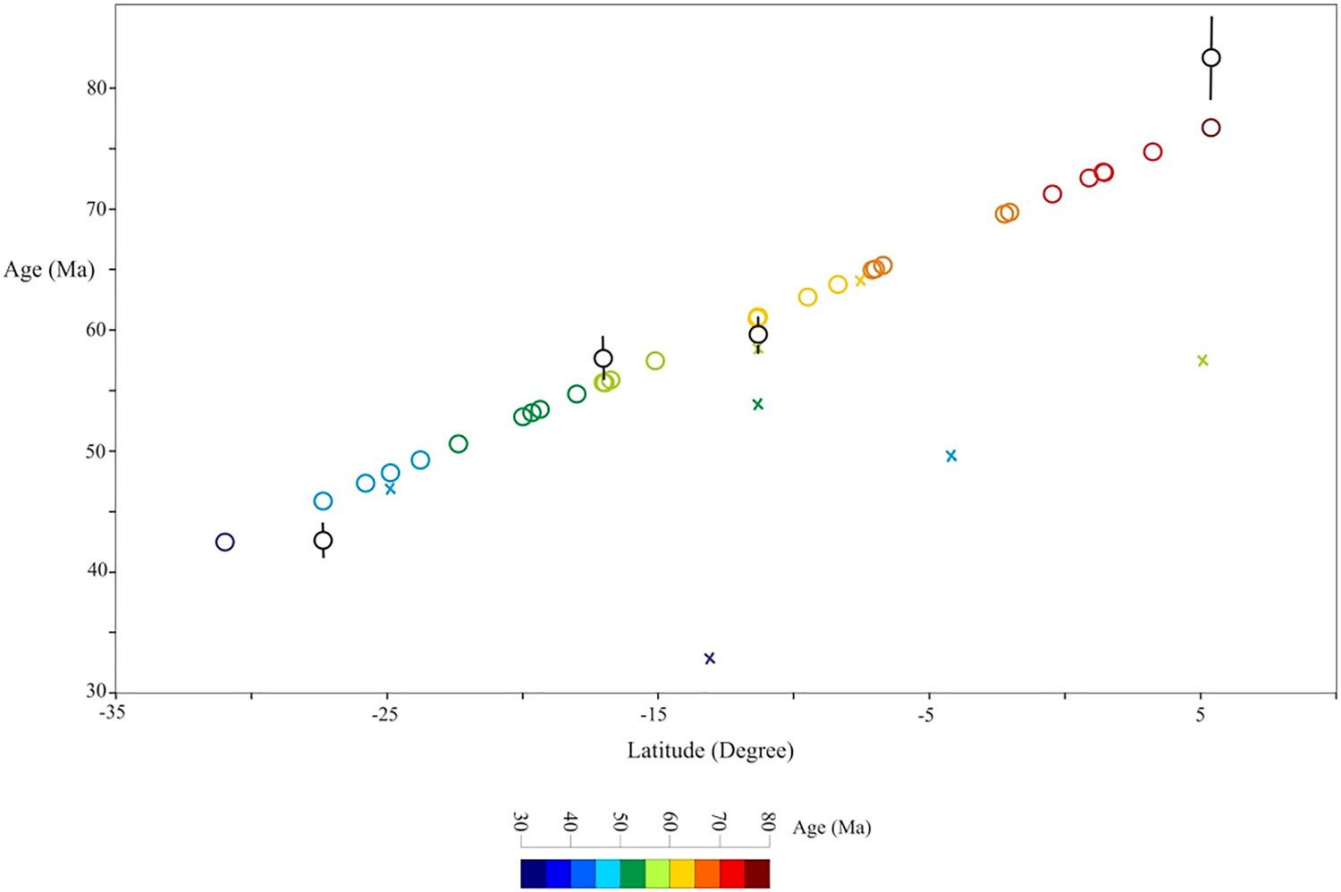
The Ninetyeast Ridge basalt ages based on the linear age propagation equation of Pringle et al.^49^, [Age = 0.9423 × Latitude + 71.67]. Black circles with 2-sigma error are absolute ages^63^; the absolute ages and linear age propogation line are highly correlated at the middle part of the NER including the decoupling event at ca. 55 Ma. Colored crosses are samples from Chagos-Laccadive Ridge and the Mascarene Plateau.

The NER samples were recovered during the DSDP, ODP, and 2007 KN0X06 cruises. Most of the samples are tholeiitic and alkaline basalts, except perhaps for the oceanic andesite recorded from site 214^56^. The NER basalts are enriched in incompatible elements like OIB, and are similar to those of the Kerguelen Archipelago^36,54,60,61^. Post-magmatic alteration processes have affected the NER basement rocks^56,61,62^, hence we used a selection of incompatible elements which are insignificantly affected by alteration, and isotopic signatures to avoid using the sensitive mobile elements for post-magmatic low-temperature alteration. Absolute age data for the NER are sparse, so ages were calculated based on the linear age propagation equation of Pringle et al.^49^, where [Age = 0.9423 × Latitude + 71.67]. Meanwhile, there’s a deviation between Pringle et al.^49^ equation, and the published absolute ages, but both are highly correlated at ca. 55 Ma (Fig. 2).

### Trace elements composition

**Figure 3:**
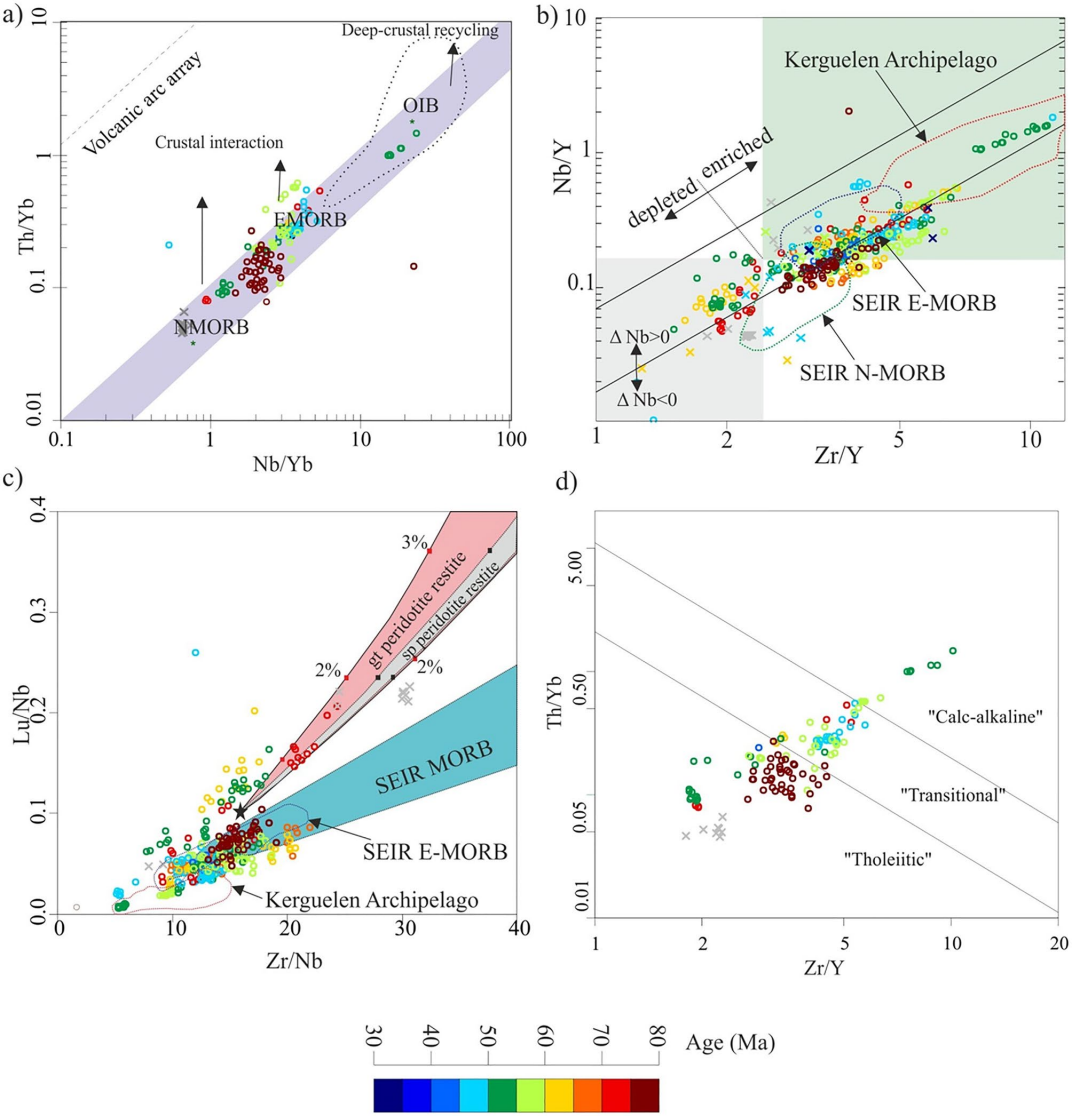
Non-subducting MORB-OIB arrays. (**a**) Th/Yb vs. Nb/Yb^57^ to discriminate between MORB–OIB, and volcanic arc arrays. The NER basalts are enriched basalts, with less crustal interaction, except perhaps for ca. 55 Ma basalts, which encountered extensive deep crustal recycling. (**b**) Nb/Y vs. Zr/Y array^58,66^ is used to distinguish between enriched/depleted Iceland basalt, and the Atlantic MORB; the NER basement rocks are similar to the Icelandic basalts^54^ with Δ Nb > 0. (**c**) Garnet/clinopyroxene Lu/Nb vs. Zr/Nb sensitive ratios^55^; melting trajectories based on Zou^68^ equations, and calculated supposing 2% porosity^55^. (**d**) Th/Yb vs. Zr/Y immobile elements discrimination diagram to determine the magma type^59^. Colored circles are the NER basalts, assigned to their ages based on the colored ages bar; the colored crosses are samples from the Chagos-Laccadive Ridge and the Mascarene Plateau, while grey crosses are not assigned to ages.

Th–Nb–Yb proxy is sensitive to mantle enrichment along MORB–OIB array, whereas higher Th/Yb values that displaced the non-subducting array to subducting (arc) array involves addition by subduction components^57^. The enrichment in Th for the MORB–OIB array could be related to deep crustal recycling; NER samples plot within the MORB–OIB array with both depleted and enriched basalts supporting non-subduction tectonic processes (Fig. 3a), and confirm plume–ridge related processes. The mantle was enriched abruptly in incompatible elements, such as Nb, Th, Zr once at ~ 55 Ma, whereas depleted basalt erupted contemporaneously, and this assured the presence of two sources for the magmatism along the NER (Fig. 3a). The interaction between the SEIR and KMP lasted for the whole lifespan of the NER, and produced depleted/enriched MORB, except perhaps for the decoupling event at ca. 55 Ma.

Niobium (Nb) as a proxy is a key discriminator between different mantle reservoirs relative to other incompatible elements^64^, hence Nb–Zr–Y discrimination diagram compare non-subducting basalts to Icelandic plume volcanics^58,65^. The NER basalts are similar to those of Icelandic magmatism, where both depleted and enriched basalt varieties exist^54^. In this connection we apply delta (Δ) Nb as a parameter to discriminate between MORB and OIB, where Δ Nb = 1.74 + log (Nb/Y) − 1.92 log(Zr/Y)^58,66^. Δ Nb = 0 line divides the field into Nb depleted (Δ Nb < 0), below that line, and Nb enriched (Δ Nb > 0) (Fig. 3b); NER basalts have both enriched and depleted affinities. Enriched basalts in that diagram exhibit positive Δ Nb anomaly, and can be interpreted to be associated with the mantle plume^65,67^.

The enriched magmatism of NER plot in three different groups, slightly enriched pertain to SEIR N-MORB, moderately enriched plot in the field of SEIR E-MORB, and highly enriched basalts with affinity similar to the Kerguelen Archipelago (Fig. 3b). Extremely enriched basalts are related to the ca. 55 Ma group, whereas depleted to enriched varieties also erupted contemporaneously. However, NER samples plot mainly in SEIR MORB with both enriched and depleted affinities, and in the Kerguelen Archipelago field, some samples are enriched in Lu (Fig. 3c), which is accompanied with enrichment in Y and Sc^55^. Lu, Y, and Sc are compatible in garnet, and their enrichment at a given Zr/Nb could be generated by partial melting of either garnet- or spinel-bearing peridotites^55^.

The discrimination between subalkaline magmas is based on combinations of the incompatible trace elements Th, Yb, Zr and Y. Thus, in the diagram of Th/Yb vs. Zr/Y diagram^59^, most samples are of tholeiitic character, whereas others plot in the transitional field between tholeiitic and calc-alkaline fields. The ~ 55 Ma basalts are the only samples that exhibit calc-alkaline behavior, whereas the tholeiitic type also exists, but without samples of transitional character (Fig. 3d). The existence of bimodal (tholeiitic/calc-alkaline) basalts occurs simultaneously at 55 Ma, confirming the decoupling between the sources of melts, and suggests that the enrichment related to deep crustal recycling is induced by continental crust abrupt recycling that is linked to the initiation of India–Eurasia collision, then basalts became transitional.

**Figure 4:**
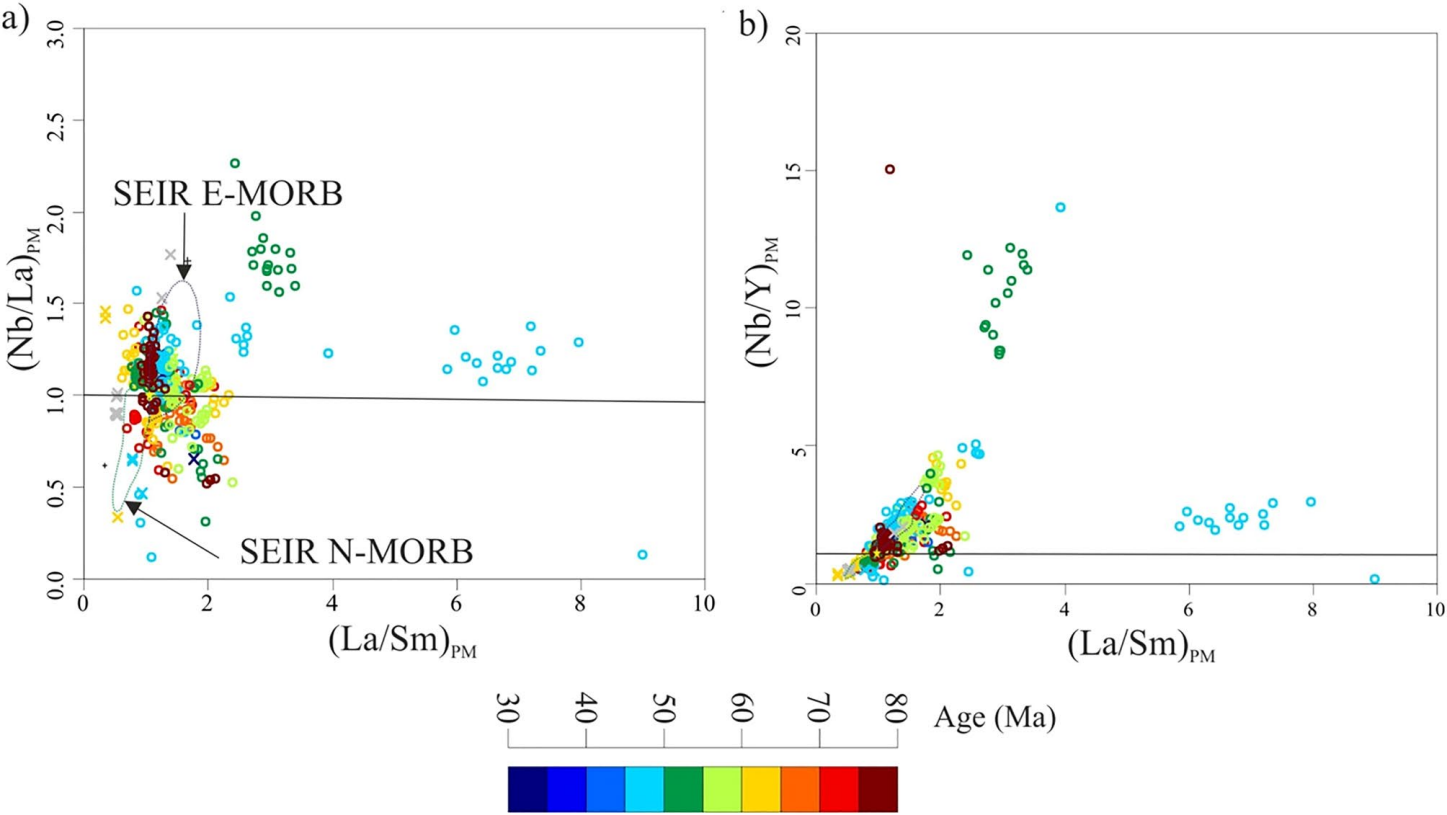
(La/Sm)_PM_ vs. (Nb/La)_PM_ and (Nb/Y)_PM_ normalized to primitive mantle^64^; where (Nb/Y)_PM_ = 1 and (Nb/Y)_PM_ = 1 at given (La/Sm)_PM_, are used to discriminate between SEIR N-MORB and SEIR E-MORB^54^.

Normalized La/Sm ratios to primitive mantle^64^ can effectively remove the effect of magma differentiation processes, where (La/Sm)_PM_ sets SEIR E-MORB apart from SEIR N-MORB^54^ at (Nb/La)_PM_= 1, and (Nb/Y)_PM_ = 1. The NER magmatism has both the depleted and enriched affinities, whereas ca. 55 Ma magmatism shows Nb enrichment abnormally than any other NER magmatism (Fig. 4a,b), and this positive anomaly in Nb is considered as a key indicator for mantle enrichment^65^, that could be related to deep recycling of crustal materials^69^. The post-collision enrichment in La (50–45 Ma), could be related to the transition to less compressional regimes, that decrease the pressure and favor the partial melting of spinel peridotite than garnet peridotite (Fig. 4).

### Isotopic signature

**Figure 5:**
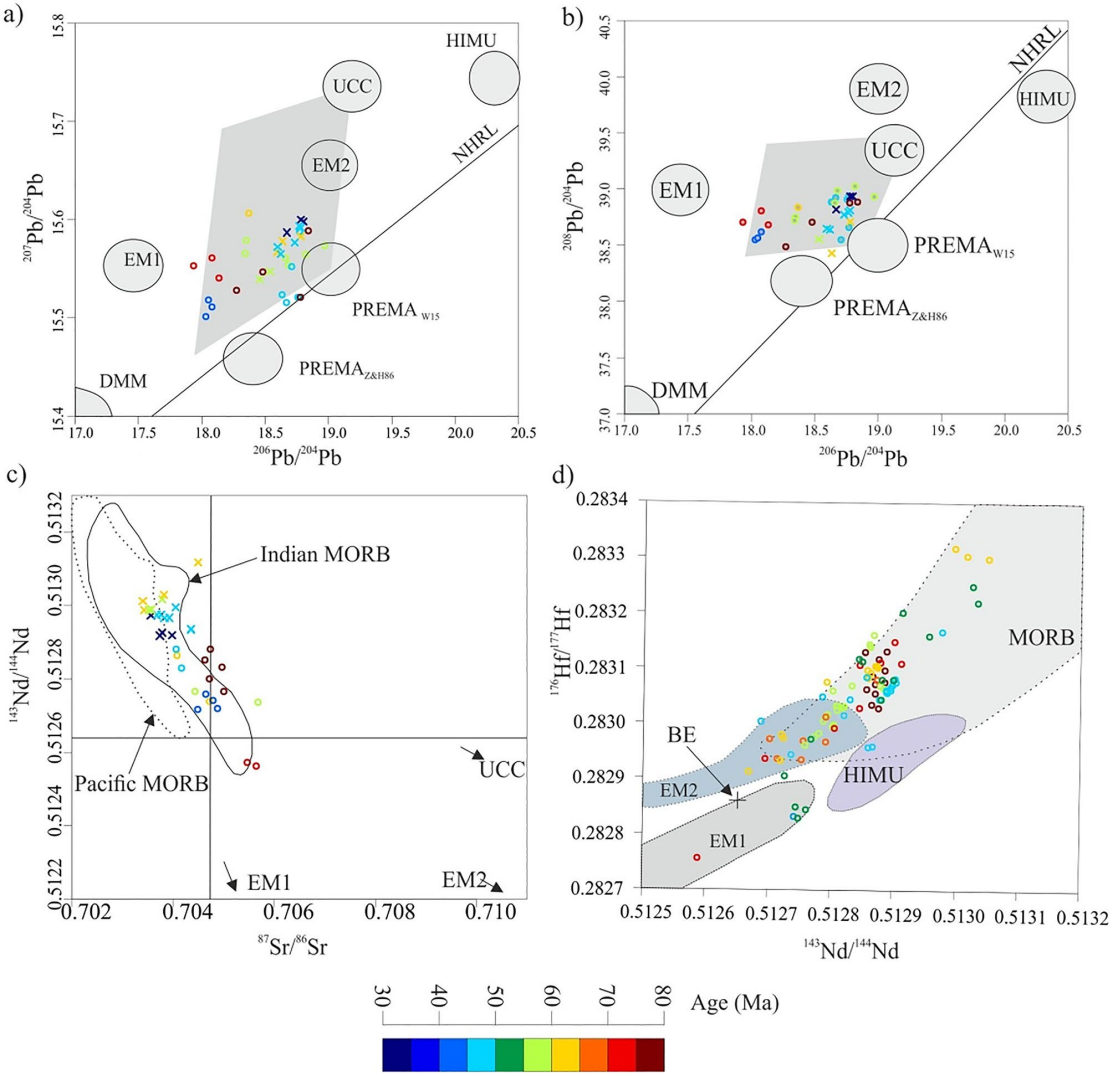
The isotopic signature of the NER basalts. (**a,b**) ^207^Pb/^204^Pb vs. ^206^Pb/^204^Pb and ^208^Pb/^204^Pb vs. ^206^Pb/^204^Pb; above the NHRL defines the DUPAL anomaly, enriched mantle 1 (EM1), enriched mantle 2 (EM2), upper continental crust (UCC), high U/Pb (HIMU), the prevalent mantle (PREMA_W15_) and (PREMA_Z&H86_) proposed by White^69^ and Zindler and Hart^73^ after Doucet et al.^71^, and depleted mantle (DMM). The NER basement rocks are similar to the deep African OIB, and plotted in the field of mixed PERMA_W15_ (prevalent mantle) + UCC (upper continental crust)^71^. (**c**) ^143^Nd/^144^Nd vs. ^87^Sr/^86^Sr, the NER basalts plot in the Indian MORB field. **d**, ^176^Hf/^177^Hf vs. ^143^Nd/^144^Nd, ca. 55 Ma basalts plot in two contrasting fields EM1 and MORB.

The southern hemisphere basalts have anomalously high ΔSr = [^87^Sr/^86^Sr − 0.7030] × 10^4^], ^208^Pb/^204^Pb, and ^207^Pb/^204^Pb isotopes, an anomaly known as DUPAL^70^; this is controlled by the two large low-shear velocity provinces underneath the Pacific and Africa, mantle sole domain with low S-wave velocity, and does not rely on the southern hemispheric basalt classification^71^. The KMP is fed from the lower mantle^19,71^, and pertains to the African mantle domain^71^. The African and Pacific mantle domains structure is linked to be in dynamic relationship with tectonics^71^, therefore, the interaction between the SEIR and the KMP along the NER reflects the relationship between deep mantle geochemical state and plate tectonics. The NER basalts have higher ^207^Pb/^204^Pb, and ^208^Pb/^204^Pb isotope ratios (Fig. 5a,b), and plot above the NHRL line that defines the DUPAL anomaly, and in the field of mixed PERMA_W15_ (prevalent mantle) + UCC (upper continental crust)^69^ and this confirms their lineage to the African mantle domain (Fig. 5 a&b). The NER magmatism has higher Sr isotope values than the Pacific MORB (Fig. 5c), where significant crustal materials were recycled back, including sediments^69^. The ca. 55 Ma anomaly plot in the EM1 field based on ^176^Hf/^177^Hf vs. ^143^Nd/^144^Nd isotope diagram (Fig. 5d), and this could be attributed to the involvement of crustal materials, including sediment recycling^69^. Based on the isotopic composition of the NER magmatism, these melts were derived from enriched mantle, as a result of recycling crustal materials. The removal of the roots of the Indian plate during the breakup of Gondwana supercontinent, as a result of warming up the lithosphere by mantle plume^72^, enriched the mantle by incompatible elements underneath the Indian Ocean. However, the abrupt enrichment at ~ 55 Ma was related to another deep crustal recycling event, and this event coincided with the abrupt slowdown in the velocity of the Indian plate, most probably related to the initiation of India–Eurasia collision.

## MOR–plume interaction cycles

### Long-term coupling cycles

The longevity of interaction between the SEIR and KMP continued for a long period^54^. During the time period from 77 to 32.9 Ma, basaltic magma was generated from enriched mantle similar to the main type extruded along the NER, with E-MORB, N-MORB and OIB affinities (Fig. 3). The diversity in basalt types confirms variations in mantle sources for melts^54^, and most probably changes in the behavior of ridge–plume interaction related to tectonic events. The SEIR jumps southward toward the KMP occurred more frequently beneath the NER^20^, leaving fossil ridges behind^21^. Large spreading jump events occurred at 65 Ma and 42 Ma, whereas smaller jump events happened repeatedly^20^. Ridge jump mechanism of interaction between the SEIR and KMP, increased the longevity of the interaction and stability of the SEIR system. Therefore, the SEIR–KMP coupling produced both enriched and depleted melts^54^, with the involvement of crustal materials (Figs. 3, 5). These long-term coupling cycles existed more frequently during the interaction between the SEIR and KMP, but disturbed once at 55 Ma, where the mantle became more enriched abruptly, as a result of deep recycling of crustal materials (Fig. 3).

### Short-term decoupling cycle

The asymmetrical spreading of the Indian Ocean^74,75^ caused by the eastward flow of the asthenosphere^15^ is driven by MOR–plume interactions^19^. The plume–ridge interaction depends on the interaction distance, and the spreading rate of the MORs^76–78^. The interaction distance between the MOR and mantle plumes range from hundreds of kilometers^79^, to greater than 1000 km^19,22,80^. Meanwhile, perpendicular and radial structures related to the interaction between the MOR and mantle plume have been detected by seismic tomography^81^, reproduced by numerical modelling^82,83^ and analogue experiments^84^. Therefore, the off-axis mantle plume within the interaction field distance is still able to interact with the distal MORs^85^. Meanwhile, the flow of melts from the off-axis plume toward the ridge produces elementary depleted, but isotopically enriched N-MORB^77^. The migration of SEIR southward, slow spreading rate, and ridge jumps frequently occurred underneath the NER enabled long-term interaction between the SEIR and KMP^86^. The second enrichment event at ~ 55 Ma along the NER produced the OIB and E-MORB separately (Fig. 3a,b). Meanwhile, the Nb anomaly of these events assure the deep crustal recycling related to plume–ridge interaction (Fig. 3a,b). Moreover, immobile trace elements discrimination of ~ 55 Ma anomaly basalts into tholeiitic and calc-alkaline types, confirms the geochemical decoupling, that is different from the off-axis plume (Fig. 3d). The existence of two contrasting rock types being formed, including OIB (Fig. 3), and the isotopic signature of the second enrichment event with low ^143^Nd/^144^Nd ratio confirms the interaction between SEIR and the on-axis KMP at ~ 55 Ma event. Therefore, the geochemical decoupling of the SEIR and the KMP occurred while the KMP was on-axis, and this reflects profound changes in the chemical properties of the mantle. Meanwhile, the abrupt slowdown in the Indian plate drift at 55 Ma can be explained to coincide with the beginning of India–Eurasia collision^7,17^.

## Discussion

The interaction between mantle plumes and nearby MORs controlled the production rate of magma fluxes^87^, and plume–MORs interaction magnitude is a function of mantle temperature^88^ and the spreading rate^89^. Thus, in return any change in mantle geochemistry could affect the plume–MORs interaction activities. Consequently, the diversity in melts extruded along MORs records variations in mantle sources for melts^54^, and most probably changes in the behavior of ridge–plume interaction related to tectonic events. The basalts erupted along the Indian Ocean ridges are elementary and isotopically of different composition compared to both the Atlantic and Pacific MORBs^90^ (Fig. 5), because of their interaction with plumes feeding from the African large low-shear velocity province mantle domain^71^. However, SEIR is a fast migrating MORs^19^, but the interaction of KMP and the SEIR persisted for a long time^54–56^, a process induced by the tectonic ridge jumps^20,21^. Meanwhile, the KMP is considered as a deep plume, feeding from the lower mantle^19^, so NER magmatism related to KMP–SEIR conjunction is the product of interaction between shallow and deep mantle reservoirs. Therefore, the first enrichment event for the mantle underneath the Indian Ocean that produced enriched basalts was induced by the large low-shear velocity province of the African mantle domain^71^, and this effect persisted for the lifespan of the NER. Simultaneously, the elimination of the Indian plate roots as a result of Gondwana supercontinent dispersal^72^, enriched the mantle underneath the Indian Ocean. Therefore, Precambrian chunks of continental material were found in basaltic rocks in the Indian Ocean, such as garnet granulite xenoliths that found in the basaltic basement of Elan Bank^36,37^, and inherited Archaean zircon recorded from Mauritius Island Miocene lava^38^.

**Figure 6:**
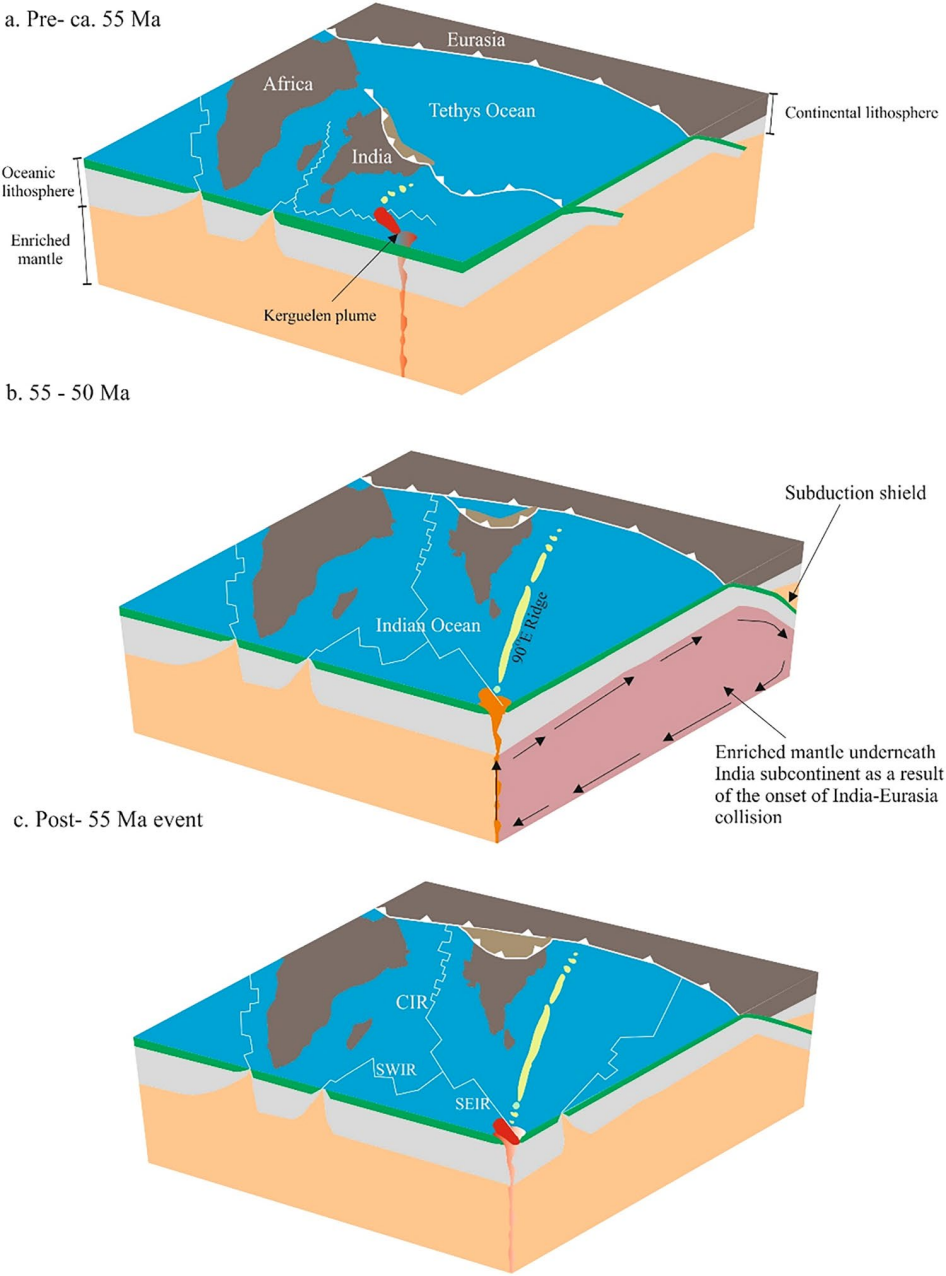
MOR–plume interaction cycles. (**a**) MOR–plume coupling, slightly enriched mantle related to identical interaction between the KMP and the SEIR produced E-MORBs. (**b**) Decoupling of MOR–plume at ca. 55 Ma; the onset of India–Eurasia collision led to continental materials recycling into the mantle. (**c**) Recoupling of MOR–plume postdates India–Eurasia collision.

The onset of India–Eurasia collision led to quiescence in the Neotethys closure activity, and low spreading rates along the Indian Ocean ridges^18^, thus causing deceleration in the northward drift of the Indian plate^91,92^. The subduction zones influenced the mantle by recycling crustal materials, including sediments^69,93^, hence modifying the asthenosphere mantle^94^, then recycling it back into MORs^94,95^, and into arc magmatism^93^. A group of microcontinental blocks existed between India and Eurasia before the closure of the Neo-Tethys^96^, this is a common phenomenon at many continental margins^97^, and was induced by mantle plume^98,99^. The subduction of Neo-Tethys microcontinents before the onset of India–Eurasia collision plate affected the arc magmatism in Ladakh^100,101^. Subduction zones act as a shield that divide the mantle tectonically and prevent the convection of enriched mantle between different domains laterally^94^. Consequently, the Neotethys northward double-subduction zones prevented the enriched mantle underneath the India Ocean from northward migration below the Tibet–Himalaya orogeny (Fig. 6).

The second enrichment event happened abruptly at ca. 55 Ma, whereas depleted and enriched basaltic rocks erupted contemporaneously, and this assures the presence of distinct sources for the magmatism along the NER derived from different mantle reservoirs (Fig. 3). However, NER samples plot mainly in SEIR MORB, some samples are enriched in Lu at a given Zr/Nb (Fig. 3c). This is correlated with enrichment in Y and Sc^55^, and could give evidence for the partial melting of either garnet- or spinel-bearing peridotites^55^. However, ca. 55 Ma basalts have both tholeiitic and calc-alkaline affinities without transitional samples (Fig. 3d), indicating the presence of immiscible distinct sources. These distinct sources exist along the NER, giving both enriched and relatively depleted rock types that mixed homogeneously, but the rapid enrichment related to the second phase boost the decoupling into distinct rock types contemporaneously. Therefore, the second enrichment event was related to deep crustal recycling, that is induced by continental crust abrupt recycling, and this is linked to the initiation of India–Eurasia collision. Most of the collisional age estimation methods are biased; the stratigraphic age-constraints are mostly older, while tecto-magmatic ages are younger (Fig. 1a). The abrupt slowdown in the Indian plate set the onset of India–Eurasia collision to be at 55 Ma^7,18,44^, and this is consistent with the SEIR–KMP decoupling event related to the deep recycling of crustal materials underneath the Indian Ocean. However, the break-off of the Neo-Tethys subducted slab ca. 53 Ma is explained as the reason behind the slowdown in the drift of the Indian plate, rather than India–Eurasia collision^18^. The onset of India–Eurasia collision preceded the break-off of Neo-Tethys subducted slab break-off; Zhu et al.^18^ set ca. 55 Ma to be the time for India–Eurasia collision initiation.

The NER magmatism has both the depleted and enriched affinities, whereas ca. 55 Ma magmatism shows Nb enrichment abnormally compared with any other NER magmatism (Fig. 4a,b), and this positive anomaly in Nb is considered as a key indicator for mantle enrichment^65^, that could be related to deep recycling of crustal materials^69^. The post-collision enrichment in La (50–45 Ma), could be related to the transition to less compressional regimes, that decrease the pressure and favor the partial melting of spinel peridotite rather than garnet peridotite (Fig. 3c). The NER basalts have higher ^208^Pb/^204^Pb, and ^207^Pb/^204^Pb isotope ratios (Fig. 5a,b), and plot above the NHRL line that defines the DUPAL anomaly, and in the field of mixed PERMA_W15_ (prevalent mantle) + UCC (upper continental crust)^69^ and this confirms their lineage to the African mantle domain (Fig. 5a,b). The NER magmatism has higher Sr isotope than the Pacific MORB (Fig. 5c), where significant crustal materials were recycled back, including sediments^69^. The ca. 55 Ma anomaly plot in the EM1 field based on ^176^Hf/^177^Hf vs. ^143^Nd/^144^Nd isotope diagram (Fig. 5d), and this could be attributed to the involvement of crustal materials, including sediment recycling^69^. Based on the isotopic composition of the NER magmatism, these melts were derived from enriched mantle, as a result of recycling crustal materials.

The earlier enrichment of the mantle underneath the Indian Ocean was triggered by the African large low-shear velocity province domain^71^. Meanwhile, the Indian plate lost the lower part of its lithosphere during the breakup of Gondwana^72^, with evidence for recycling of the Indian lithosphere in the basaltic basement of Elan Bank^36,37^, and in Mauritius Island Miocene lava^38^ (Fig. 6a). However, two-stage diachronous collisions happened^13^, but the disturbance in MOR–plume decoupling happened once at ca. 55 Ma (Fig. 6b), and this was coincident with the second enrichment event (Fig. 6b). The disturbance in MOR–plume interaction led to on-axis decoupling between MOR–plume, as a result of deep recycling of lithospheric materials. Consequently, the geochemical composition of the mantle changed, and this is most probably related to the collision between India and Eurasia. E-MORBs extruded predominantly to postdate the collision of India–Eurasia, thereafter MOR–plume reconciled (Fig. 6c). The interaction of MOR–plume is very sensitive to major geodynamic events, such as India–Eurasia collision, and could be used to make the timing of geologic events more precise.

## Methodology

A geochemical database of non-subducting-influenced basaltic rocks, consisting mainly of E-MORB and OIB in the Indian Ocean especially along the NER, Chagos-Laccadive ridge, and Mascarene Plateau, has been analyzed (Supplementary Table 1) and applied for the present study. The integrated Sr, Nd, Pb, Hf isotope, and trace elements (particularly actinide elements such as Th, transition elements such as Nb, Y, Lu, and Zr, in addition to lanthanide elements such as La, Yb, and Sm) of basalts (6550 samples), with ages ranging from 77 to 32.9 Ma, were retrieved from the EarthChem repository. Data reduction was applied using the Pandas–Python data analysis library in Jupyter notebook IDE in order to exclude samples with abnormal values. After an automated check based on the Pandas library, a manual double-check was carried out. Out of 6550 samples, 1164 were used in this study and plotted on the map to cross-check the age of the samples relative to their location along large igneous province ridges (Fig. 1b), based on the results from Refs.^19,102^. 5386 samples were excluded from this study, because they are located outside the NER and/or reduplicated. The geochemical data of basalt volcanisms extruded along the NER are available from https://www.earthchem.org/, and were reduced using Jupyter (https://jupyter.org/), and plotted using the GeoChemical Data Toolkit software (GCDkit http://www.gcdkit.org/).

## Supplementary Information

Supplementary Table 1.

## Data Availability

All data generated or analysed during this study are included in this published article and its Supplementary Information files. Figures 1a and 6 were generated using CorelDRAW software, and Fig. 1b generated using GPlates.
